# Supplementary figures and images for: Full-Length Transcriptome Characterization and Functional Analysis of Pathogenesis-Related Proteins in Lilium Oriental Hybrid ‘Sorbonne’ Infected with Botrytis elliptica
Source: Int J Mol Sci. 2022 Dec 27;24(1):425. doi: 10.3390/ijms24010425 (PMC9820132; doi:10.3390/ijms24010425)

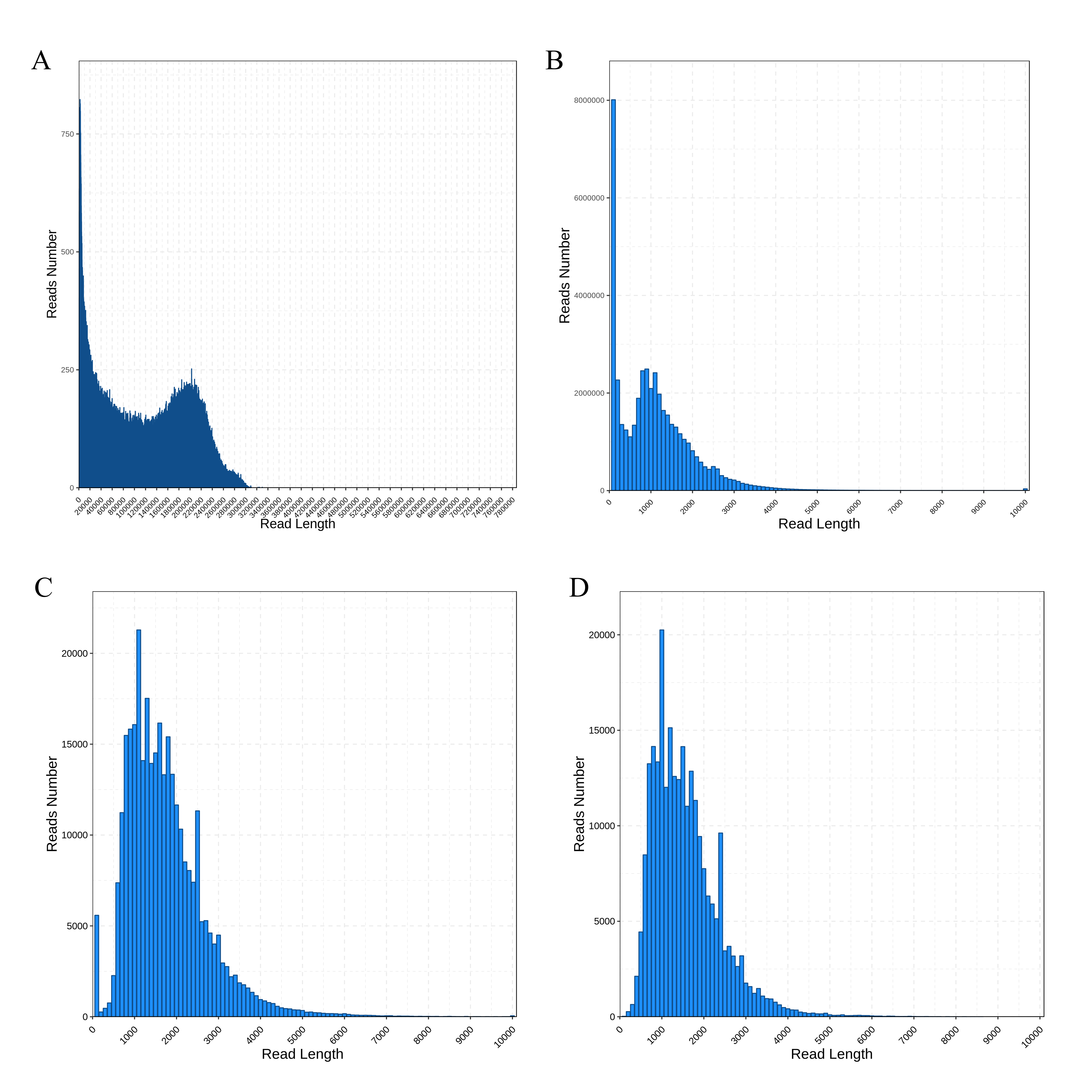

Supplement: Supplementary file 1 [file ijms-24-00425-s001.zip › Supplementary Figure S1.tif]

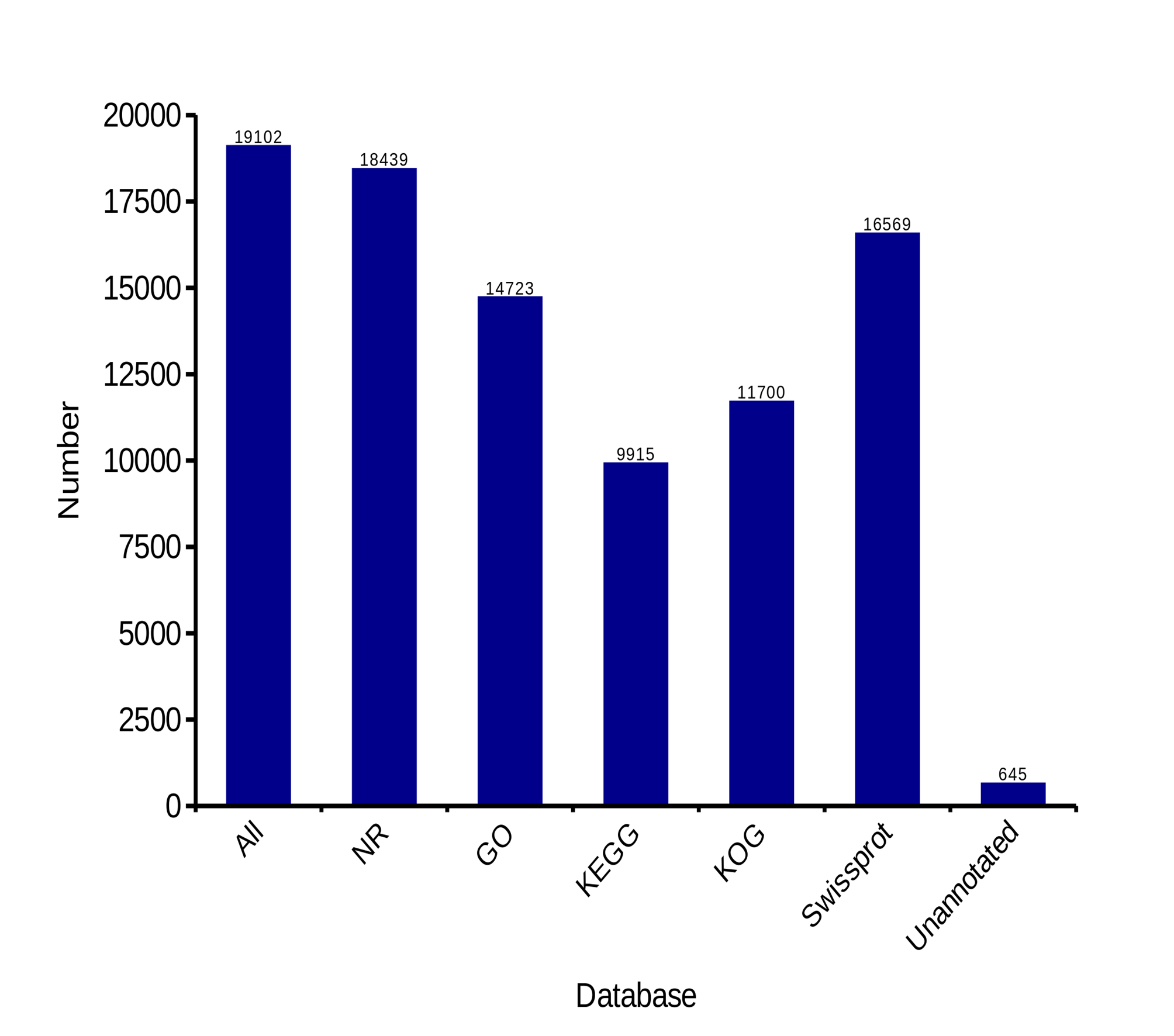

Supplement: Supplementary file 1 [file ijms-24-00425-s001.zip › Supplementary Figure S2.tif]

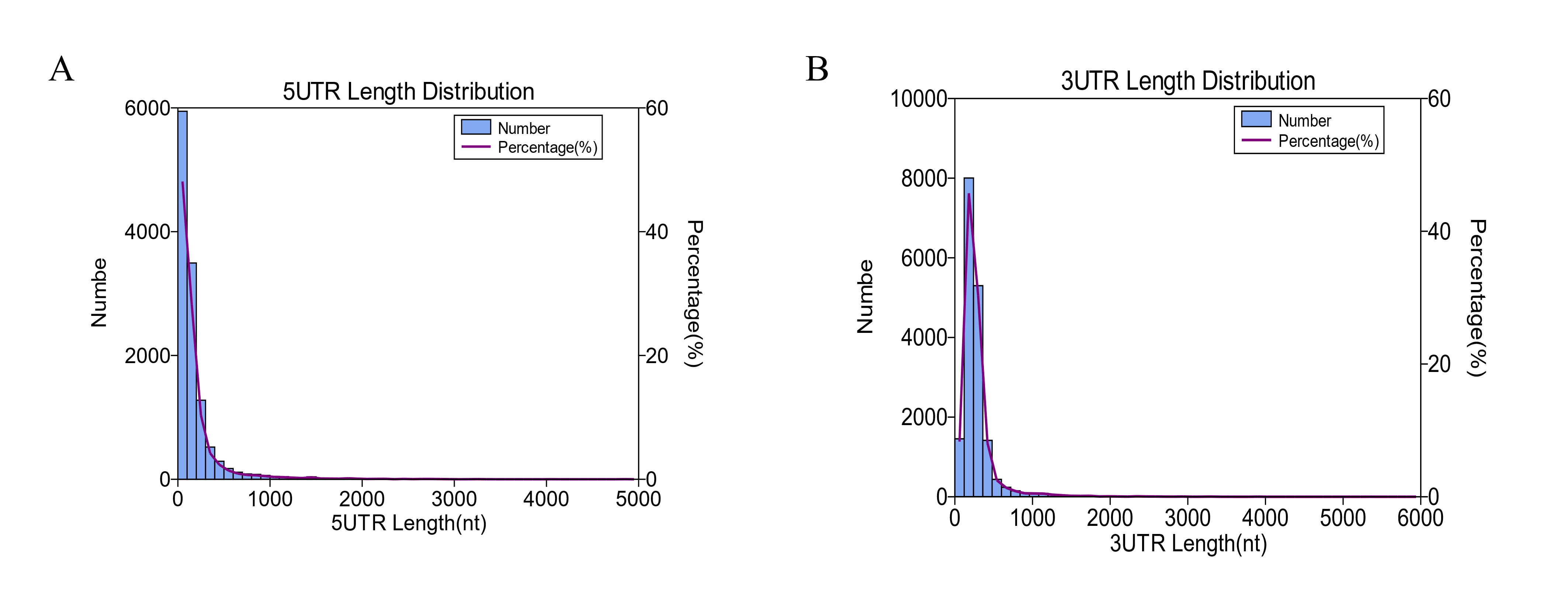

Supplement: Supplementary file 1 [file ijms-24-00425-s001.zip › Supplementary Figure S3.tif]

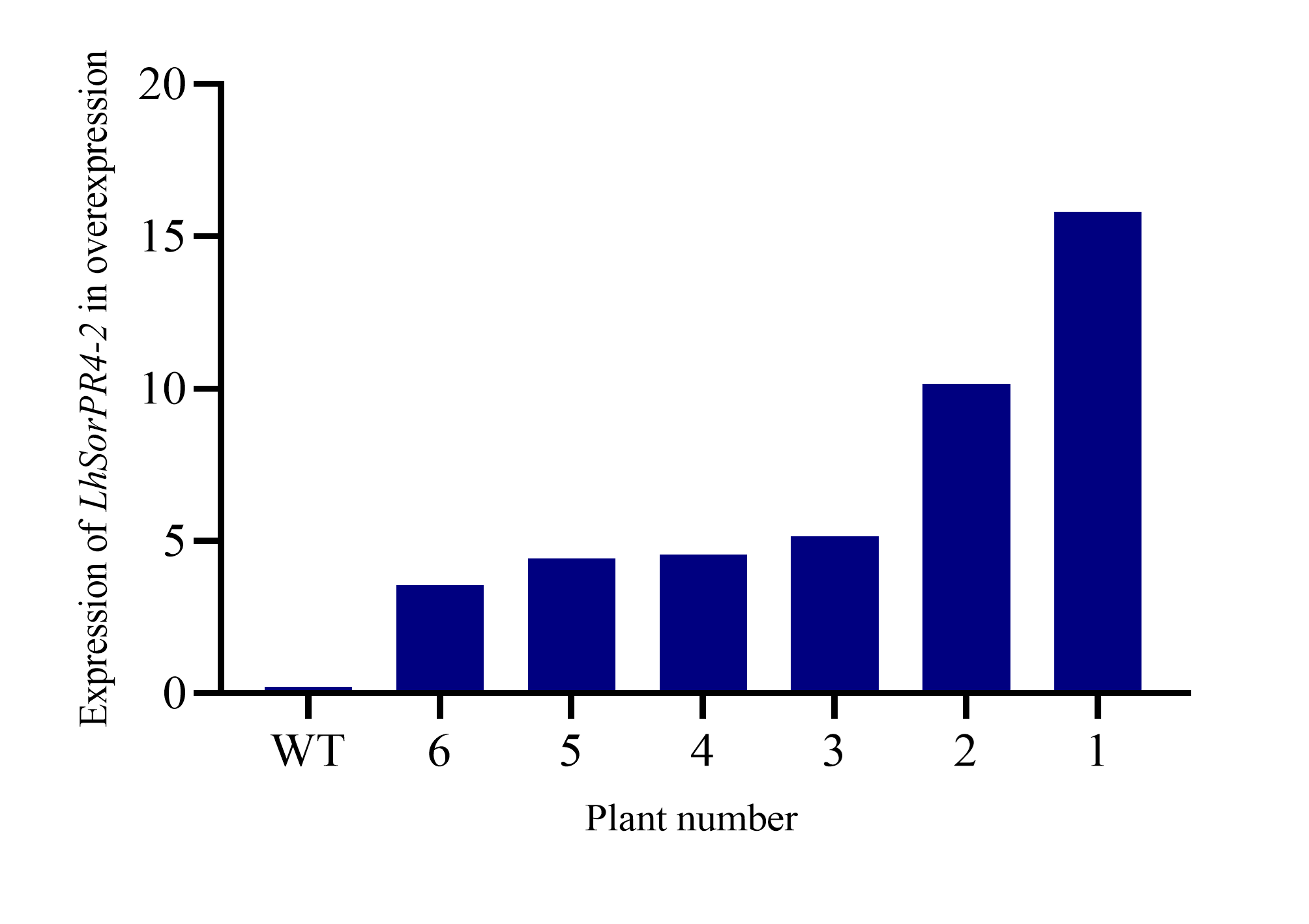

Supplement: Supplementary file 1 [file ijms-24-00425-s001.zip › Supplementary Figure S4.tif]
